# A comprehensive strategy to detect the fraudulent adulteration of herbs: The oregano approach

**DOI:** 10.1016/j.foodchem.2016.05.004

**Published:** 2016-11-01

**Authors:** Connor Black, Simon A. Haughey, Olivier P. Chevallier, Pamela Galvin-King, Christopher T. Elliott

**Affiliations:** Institute for Global Food Security, Advanced ASSET Centre, School of Biological Sciences, Queen’s University Belfast, Northern Ireland, United Kingdom

**Keywords:** Oregano, Authenticity, Adulteration, Fourier transform infrared, High resolution mass spectrometry, Biomarkers

## Abstract

Fraud in the global food supply chain is becoming increasingly common due to the huge profits associated with this type of criminal activity. Food commodities and ingredients that are expensive and are part of complex supply chains are particularly vulnerable. Both herbs and spices fit these criteria perfectly and yet strategies to detect fraudulent adulteration are still far from robust. An FT-IR screening method coupled to data analysis using chemometrics and a second method using LC-HRMS were developed, with the latter detecting commonly used adulterants by biomarker identification. The two tier testing strategy was applied to 78 samples obtained from a variety of retail and on-line sources. There was 100% agreement between the two tests that over 24% of all samples tested had some form of adulterants present. The innovative strategy devised could potentially be used for testing the global supply chains for fraud in many different forms of herbs.

## Highlights

• Two tier strategy proposed to detect oregano fraud.
• FT-IR screening and HR-LC-MS confirmatory methods developed.
• Unique biomarkers discovered in adulterants by HR-LC-MS.
• Chemometric calibration models generated.
• 24% of oregano samples tested in UK/Ireland were found to be adulterated.

## 1 Introduction

Globally, herbs and spices play a significant part in the diets of many as they are important ingredients in a multitude of foods, beverages, medicines and cosmetics. With consumers having greater access and a desire to use these products, the demand has increased vastly over the last thirty years making it a multibillion dollar industry (Furth & Cox, 2004). Marieschi, Torelli, Poli, Sacchetti, and Bruni (2009) stated that the global herb and spice trade was worth $2.97 billion with the EU market amounting to 520 thousand tonnes and a value of €1.8 billion. Sales in 2014 at all United Kingdom (UK) supermarkets were £173 million for dried herbs and spices, and £107 million for fresh herbs (spices not included) (Nielsen, 2015). Economically motivated adulteration (EMA) of food is a common concept, which has occurred within the food industry since trading began (Spink & Moyer, 2013). As is the case with any food commodity, there is a greater possibility of food adulteration when the demand and prices increase and when complex supply chains are involved. Herbs and spices fulfil all this criteria.

Herbs and spices are two very different food items with spices tending to be bright vibrant colours emanating often from warm climates in a diverse form e.g. cumin and turmeric and herbs are usually green leaved and derived from plants in cooler environments e.g. parsley and thyme. Supply and demand is a fundamental economic principle which determines the price of all commodities. However, as well as this, the price of spices is also dictated by the intensity of their colours and therefore, common adulteration of spices had been the addition of illegal dyes such as Sudan dyes, which are group 3 genotoxic carcinogens (Cornet et al., 2006, Ruf et al., 2012). However, since this issue was highlighted in 2003, the addition of Sudan dyes as a food additive has been banned worldwide. Herbs are not traded on colour and so there is no economic advantage gained from adding dyes. Instead, the price of herbs is dependent on how compact the product is and therefore bulking agents have been used as most commercially sold herbs tend to be either chopped or ground. Consequently, it is relatively easy to add other cheaper ground bulking agents without the supply chain and indeed the consumer noticing.

Oregano is a culinary herb most commonly associated with pizzas and other Mediterranean dishes. The main producers of oregano reside in the United States of America, Mexico, Greece and Turkey. Compared to most herbs, oregano has a complicated history as the true identity of it is very difficult to define. This is partly due to the large heterogeneity of the *Origanum* genus, but also due to the grouping of different botanical genera*; Origanum* (*lamiaceae*) from the Mediterranean and *Lippia* (*verbenaceae*) from Mexico (Marieschi et al. 2009). Due to the confusion, this led to a clear market distinction between Mediterranean and Mexican oregano, with both having different cleanliness specifications such as the addition of sumac leaves (ASTA, 2015). Mexican oregano has a much stronger and robust flavour compared to Mediterranean oregano, which could be due to the varying percentages of essential oils within the leaves. The essential oil percentage in Mexican oregano leaves is around 3–4%, whilst the percentage within Mediterranean oregano leaves is around 2–2.5%.

Even with the clear commercial distinction between Mexican and Mediterranean oregano, there are still several different definitions regarding Mediterranean oregano. The European Pharmacopoeia (PhEur) and the European Spice Association only allow *Origanum vulgare L.* ssp. *hirtum* and *Origanum onites L*., to be marketed as true oregano with impurities of extraneous materials of up to 2% being considered tolerable (ESA, 2015a, ESA, 2015b, Marieschi et al., 2011a, Marieschi et al., 2011b, European Pharmacopoeia Journal, 2005). However, ISO/FDIS 7925 (2015) allow leaves of all *Origanum* genus, species and subspecies except *Origanum majorana L*, to be marketed as oregano. Impurities of up to 1% are considered tolerable, which is in line with the value accepted by American Spice Trade Association (ASTA, 2015, Nielsen, 2015).

There has been a range of different detection methods developed and used in the determination of the authenticity of food. In a review by Reid, O’Donnell, and Downey (2006) many methods of detections were scrutinised including spectroscopy (ultraviolet-visible (UV), near infrared (NIR), mid infrared (MIR), Raman), isotopic analysis, chromatography, electric nose, polymerase chain reaction, enzyme-linked immunosorbent assay and thermal analysis – all of which are techniques that have been applied to food authentication since 2001. Food fingerprinting is of particular interest as a method of detection. Ellis et al. (2012) published a review of some of the fingerprinting technologies, with particular interest paid to NIR, MIR and Raman spectroscopic techniques. Few analytical methods are available to screen a large number of dried plant materials quickly and the detection of oregano adulteration has been limited to a few techniques. Marieschi et al., 2009, Marieschi et al., 2010, Marieschi et al., 2011a, Marieschi et al., 2011b applied a random amplified polymorphic DNA (RAPD) method which has led to the development of sequence-characterized amplified region makers (SCARs) for a number of potential adulterants; *Rhus coriaria L*., *Cistus incanus L., Olea europaea L., Rubus coriaria L.*, which lack a clearly detectable essential oil profile and *Satureja montana L., O. majorana L*., which belong to the lamiaceae family. Work has also been carried out by Bononi et al., 2010, Bononi and Tateo, 2011 who have utilized liquid-chromatography mass spectrometry (LC-MS) and gas-chromatography mass spectrometry (GC-MS) to identify the presence of olive leaves in ground oregano, using oleuropein as a marker.

The aim of this study was to develop and fully validate a two-tier approach utilising Fourier-Transform Infrared spectroscopy (FTIR) and Liquid Chromatography High Resolution Mass spectrometry (LC-HRMS) to screen for and confirm oregano adulteration. When these two techniques are combined with multivariate data analysis software they have the ability to process a large number of samples (Gao et al., 2014, Osorio et al., 2014). By applying FTIR and LC-HRMS the ability to produce the world’s first comprehensive testing system of oregano adulteration was trialled. A survey of commercially available oregano samples in the UK were then undertaken to determine the current level of abuse.

## 2 Materials and methods

### 2.1 Sample collection and preparation

Samples of oregano with full provenance and traceability and a number of previously identified adulterants (olive leaves, myrtle leaves, sumac leaves, cistus leaves, hazelnut leaves), were sourced from different parts of the world. Commercially available oregano samples were purchased at various retailers including convenience shops, supermarkets and market places in the UK and Ireland. In addition samples were also purchased from online retailers a small number that were obtained from EU and non EU countries. The samples were milled to a homogeneous powder on a PM-100 Retsch Planetary Ball Mill (Haan, Germany) by weighing approximately 5 g into grinding jars and milling at 500 rpm for 5 min.

For LC-HRMS analysis, milled homogenate herb sample (0.05 g) was extracted in 2 mL of methanol/water solution (1:1, v/v), mixed for 10 min, sonicated for 15 min at maximum frequency in a water bath at room temperature, centrifuged at 10,000*g* for 10 min at 4 °C and the supernatant collected (1 mL). The supernatant was dried and reconstituted in 1.5 mL of ultra-pure water. Subsequently, the extract was filtered through a 0.22 μm Costar Spin-X Centrifuge Tube Filter (10,000*g* at 4 °C for 10 min). Filtered extracts were immediately transferred into Waters maximum recovery vials for UPLC-QTof-MS analysis.

### 2.2 Spectral data acquisition using Fourier-Transform Infrared (FTIR)

For FTIR, the milled samples were placed in the ATR sample area of a Thermo Nicolet iS5 spectrometer (Thermo Fisher Scientific, Dublin, Ireland) equipped with ATR iD5 diamond crystal and ZnSe lens and DTGS KBr detector. The slip-clutch pressure tower is applied to the sample and tightened until the correct pressure was utilised which gives more reproducible results. Each spectrum was acquired in the 550–4000 cm^−1^ range. The acquisition parameters were: number of sample scans: 32; collection length: 47 s; resolution: 4.000; levels of zero filling: 0, number of scan points: 12415; laser frequency: 11742.96 cm^−1^; apodization: N-B Strong; phase correction: mertz; number of background scans: 32; background gain: 4.0. The acquisition was repeated 3 times. Spectral data for each sample was averaged before further data processing.

### 2.3 Chromatographic and mass spectrometry conditions

Analyses were carried out on a Waters Acquity UPLC I-Class system (Milford, MA, USA) coupled to a Waters Xevo G2-S QTof mass spectrometer (Manchester, UK) with an electrospray ionisation source operating in positive or negative mode with lock-spray interface for real time accurate mass correction. Instrument settings were as follow: source temperature was set at 120 °C, cone gas flow at 50 L h^−1^, desolvation temperature at 450 °C, and desolvation gas flow at 850 L h^−1^. The capillary voltage was set at 1.0 kV in positive mode and 1.5 kV in negative mode, respectively. Source offset was 60 (arbitrary unit). Mass spectra data were acquired in continuum mode using MS^E^ function (low energy: 4 eV; high energy: ramp from 15 to 30 eV) over the range *m*/*z* 50–1200 with a scan time of 0.08 s. A lock-mass solution of Leucine Enkephalin (1 ng μL^−1^) in methanol/water containing 0.1% formic acid (1:1, v/v) was continuously infused into the MS via the lock-spray at a flow rate of 5 μL min^−1^.

The chromatographic separation was conducted on an Acquity HSS T3 column (100 mm × 2.1 mm, 1.8 μm). The column oven temperature was set at 45 °C, injection volume at 5 μL and flow rate at 0.4 mL min^−1^. Mobile phase consisted of (A) water with 0.1% formic acid and (B) methanol with 0.1% formic acid. The gradient was set as follows: 1.50 min of 99% (A) followed by a linear increase from 1% to 99% (B) over 15 min, isocratic cleaning step at 99% (B) for 2 min, then returned to initial conditions 99% (A) over 0.25 min and column equilibration step at 99% (A) for 1.25 min. Each sample was injected three times in order to assure reproducibility. Prior to all analyses 10 pooled conditioning samples were injected. For quality control pooled samples were injected at intervals of every 10 samples throughout the entire experiment to determine the chromatographic reproducibility of retention times and peak intensities (Chevallier et al., 2015, Graham et al., 2013).

### 2.4 Data processing and data analysis

Principal Components Analysis (PCA), an unsupervised technique, and Orthogonal Partial Least Squares Discriminate Analysis (OPLS-DA), a supervised technique, were used for building the qualitative models in this investigation as previously described (Haughey, Galvin-King, Ho, Bell, & Elliott, 2015).

The generation of calibration models was carried out using similar methodology as previously published (Haughey, Galvin-King, Malechaux, & Elliott, 2014). The data pre-processing included standard normal variate technique (SNV), which compensates for differences in pathlengths due to scattering effects, 2nd order derivative and Pareto scaling using the SIMCA 14 chemometric software. Spectral data was analysed between the wavenumber ranges 550–1800 cm^−1^ and 2800–3999 cm^−1^. The data generated include R2 which is an estimate of the fit of the model and Q2 which is an estimate of the predictive ability of the model and it is calculated by cross-validation. The latter is calculated by removing each 1/7th of the data in succession and building a new model on the remaining data with the omitted data predicted using this method of cross validation. Predicted Residual Sum of Squares (PRESS) is calculated by comparison with the original data with the best predictability of the model indicated by a low value. The SIMCA 14 chemometric software automatically converts PRESS into Q2 to resemble the scale of the R2 with good predictions having high Q2.

Raw data generated by the mass spectrometer were imported to Progenesis QI 2.0 software (Waters, Newcastle, UK). After data conversion to the appropriate format using a filter set at 2, data were aligned to the best pool sample selected and peak picking from 0.5–17.5 min was carried out with sensitivity set at automatic and chromatographic peak width to 0.08. The analysed spectral data were then exported to SIMCA 14 for multivariate analysis. As a quality control measure all the spectral data were Center Scaled and analysed using PCA. All pooled samples (QC) were found to be tightly clustered within the center of each representative scores plot which indicates good reproducibility of the data. Following this, all data were mean centred, Pareto scaled and grouped into Adulterant and Oregano prior to OPLS-DA. R2 (cumulative), Q2 (cumulative) and Root Mean Squared Error of cross validation (RMSECV) were used to determine the validity of the model. R2 (cum) indicates the variation described by all components in the model and Q2 is a measure of how accurately the model can predict class membership.

## 3 Results and discussion

### 3.1 FT-IR spectroscopic analysis

**Fig. 1:**
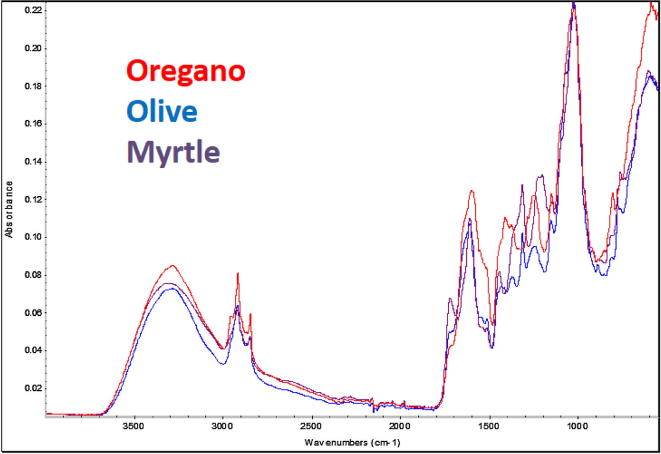
FT-IR spectra of oregano and the adulterants Olive leaves and Myrtle leaves.

**Fig. 2:**
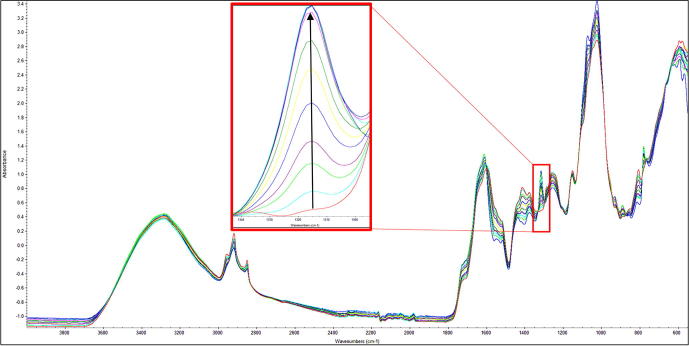
FT-IR spectra of Oregano adulterated with olive leaves in 10% additions (0–100%) showing a monotonic increase in intensity exemplified by the inset with the arrow indicating the increase in olive leaf adulteration.

**Fig. 3:**
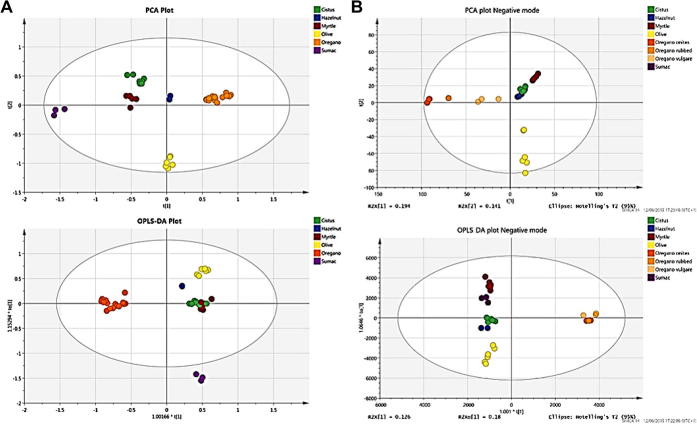
(A) Unsupervised PCA and Supervised OPLS-DA scores plots from FTIR spectral data; (B) Unsupervised PCA and Supervised OPLS-DA scores plots from LC-HRMS data in positive ionisation mode.

FTIR is a technique that is based on the absorbance of light at particular wavelengths and has been a popular methodology in detecting food adulteration. In a review by Rodriguez-Saona and Allendorf (2011), examples of using FTIR in conjunction with multivariate analysis has been applied for authentication of herbal products, fruit juices, agricultural products, edible oils, dairy, and numerous other food products. In this study, the powdered oregano and adulterant samples (olive leaves, myrtle leaves, hazelnut leaves, cistus leaves, sumac leaves) were analysed on the FT-IR spectrometer. Fig. 1 shows the FT-IR spectra of pure oregano, olive leaves and myrtle leaves. Although there was some overlap observed between the peaks in the spectrum of the oregano and the peaks in the spectra of the adulterants, visually there are observable differences in the fingerprint region from 900 to 1800 cm^−1^. FTIR peaks are attributed for stretching and bending vibrations that characterize the functional groups. The regions of interest included: (i) 1100–1400 cm^−1^, generally the most prominent peak, is due to the vibration peak of C—O in alcohol hydroxyl group (ii) 1400–1500 cm^−1^ corresponding to C—O and C—C stretching vibrations specific to phenyl groups; (ii) 1500–1600 cm^−1^ corresponds to aromatic vibrations and N-H bending and (iv) 1600–1740 cm^−1^ corresponding to bending N—H, C=O stretching (aldehydes, ketones, esters, free fatty acids and glycerides). To further observe the influence of adulterants on the spectrum of oregano, the latter was adulterated in 10% additions (0–100%) of olive leaves and the spectra recorded. Fig. 2 shows the resulting spectra indicating the monotonic increase intensity exemplified by the peak shown in the inset. Due to these differences identified in the spectral data it was possible to apply chemometric modelling for discriminant analysis. The chemometric software (SIMCA 14) was used to generate a qualitative model using PCA (unsupervised) and supervised OPLS-DA (supervised) algorithms with Pareto scaling to determine if it was possible to differentiate pure oregano from its adulterants. The data was pre-processed using SNV, 2nd order derivative algorithm with Savitzky-Golay smoothing (11 point window and 2nd order polynomial). For the unsupervised PCA model, the first four principal components describe most of the variation (84.5%) as follows: PC1 43.3%; PC2 19.4%; PC3 14.5%; PC4 7.3%. Separation was achieved mainly along PC1 and PC2 with the positive scores related to oregano samples and negative scores associated with the adulterants. The measure of fit (R2) of this PCA model was 94% and the measure of predictive ability (Q2), based on cross validation was 86%. For the supervised chemometric model, OPLS-DA was used with the same pre-processing parameters used for the PCA plot. The OPLS-DA model generated one predictive components and one orthogonal components which explained 34.8% and 23.6% of the differences respectively. The measure of prediction (Q2), based on cross-validation, was 95.9% and RMSECV = 9.7%, indicating very good predictability of the data. Fig. 3A shows PCA and OPLS-DA scores plots, and indicated that this method could be used for discriminant analysis and this approach to rapidly screen for adulteration of oregano is sufficiently robust and thus fit for purpose.

### 3.2 High resolution mass spectrometric analysis

**Fig. 4:**
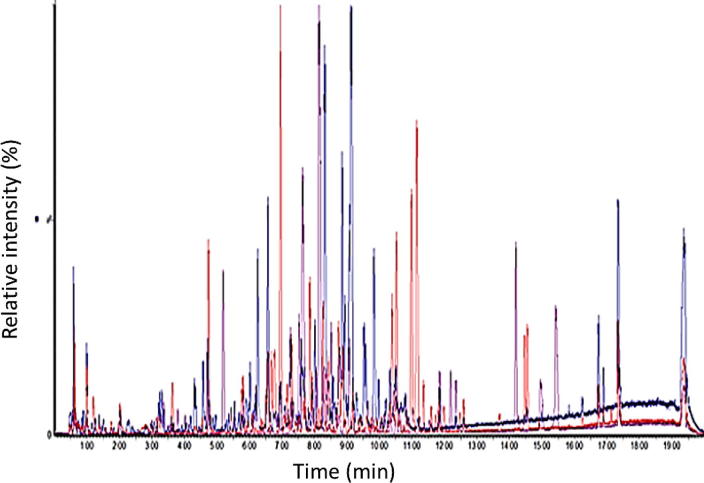
Overlay full scan chromatograms of oregano, olive leaves and myrtle leaves.

LC-HRMS is a technique that has been used extensively for metabolic profiling in both the food and plant industries (Commisso et al., 2013, De Vos et al., 2007, Masson et al., 2010). Profiling analysis of oregano and adulterant samples (olive leaves, myrtle leaves, hazelnut leaves, sumac leaves, and cistus leaves) was carried out using an untargeted analysis approach on a Waters UPLC coupled to a G2-S QT of mass spectrometer. One of the advantage of untargeted analysis by high resolution mass spectrometry in combination with chemometrics is the possibility to build models on an untargeted basis, with subsequent exploration of the data to discover the characteristic markers that contribute most significantly to the classification. Furthermore, recent improvements in instrumentation and processing software allow a faster, more reproducible and more comprehensive data analysis. In our case, up to 4500 ions in each ionisation mode were reliably detected along the chromatographic gradient (Fig. 4). The extracted data were then exported to chemometric software to be subjected to similar data treatment as was the spectroscopic data presented above. The PCA score plot generated (Fig. 3B) showed clear discrimination between the pure oregano and the adulterants, with the oregano samples clustered together on one side of the plot and the adulterant samples scattered on the other side of the plot. Additionally, there was clear separation between the two oregano species which can be marketed as Mediterranean oregano; *Origanum vulgare* and *Origanum onites*, with one sample which contained both species situated in-between the two groups. OPLS-DA was then performed and a model was generated with one latent component and three orthogonal components with resulting R2 = 99.7%, Q2 = 94.4% and RMSECV of 10.3% for the positive mode ionisation data and another one with one latent component and three orthogonal components with resulting R2 = 99.3%, Q2 = 94.9% and RMSECV of 9.4% for negative mode. Several other individual models were subsequently produce by comparing Oregano to each individual adulterant (olive, myrtle, hazelnut, cistus, sumac) with their respective S-plots to enable the potential identification of markers to each adulterant (Table 1). This methodology allowed the identification of 16 unique markers in positive mode and 12 in negative mode, with all adulterant samples having at least 4 unique markers. This data will be used for the future development of a targeted method using MS/MS analysis.

**Table 1:** Values of the statistical parameters obtained for different OPLS-DA models generated using UPLC-QT of MS data for both ionisation modes.

	Ionisation mode	Latent component	Orthogonal component	R2 (cum)	Q2 (cum)	RMSECV
Myrtle Vs Oregano	ESI −	1	1	0.994	0.984	0.062
	ESI +	1	3	0.999	0.994	0.039

Sumac Vs Oregano	ESI −	1	0	0.99	0.95	0.102
	ESI +	1	1	1	0.997	0.022

Olive Vs Oregano	ESI −	1	2	0.996	0.934	0.127
	ESI +	1	1	0.997	0.957	0.102

Cistus Vs Oregano	ESI −	1	2	0.997	0.982	0.066
	ESI +	1	5	1	0.961	0.097

Hazelnut Vs Oregano	ESI −	1	1	0.991	0.936	0.105
	ESI +	1	1	0.994	0.953	0.089

### 3.3 Survey of commercial samples

To test the models developed for both analytical methodologies, a survey of oregano was carried out which included samples from retailers, service sector, internet sources and a few bought from commercial outlets in countries outside UK/Ireland. The spectral data generated for these samples from the FTIR and LC-HRMS were predicted as unknowns using the relevant OPLS-DA model produced earlier.

Similar predictions were obtained from the models generated using both analytical techniques. Furthermore, identified markers from LC-HRMS data were found in all commercially adulterated samples and reinforce the potential of these markers for potential targeted applications. Table 2 shows the results of the survey of commercially available oregano based on the spectroscopic and spectrometric data. The samples have been broken down into those procured from the retail and service sectors in UK/Ireland and those purchased on the internet or at commercial outlets outside of UK/Ireland. The results show that approximately 24% of the oregano samples tested were adulterated and the scale of adulteration ranged from 30% to over 70%, indeed two samples had virtually no oregano present. The scale and level of the adulteration uncovered was not expected. In addition the similar figures for ‘store’ bought and ‘internet’ bought were surprising as one would have thought the retail trade would have better systems of control in place. The most common adulterants found in the samples were olive leaves and myrtle leaves. The results of the survey were passed to the regulator to alert the affected companies to the economically motivated fraud ongoing in the sector. Based on the results of this survey the samples which were indicated to be oregano by both methods can be included in the calibrations models to increase the robustness of the test.

**Table 2:** Results from the oregano survey.

Oregano Survey	UK/Ireland^a^	Internet/Other^b^
Samples Tested	53	25
Samples Adulterated	13	6
Samples Adulterated %	24.5	24
Level of Adulteration^c^	∼30 to >70%	∼30 to >70%
Most Common Adulterants	1. Olive leaves	1. Olive leaves
	2. Myrtle leaves	2. Myrtle leaves

a Includes Retail and Service Sector.

b Includes Amazon, Ebay and Purchases made abroad.

c Based on scores from chemometric analysis.

## 4 Conclusions

The detection of fraud in foods and food ingredients has become an even more important topic since the horsemeat scandal of 2013. Many consumers lost faith in the food they were purchasing and the food industry recognised that more robust measures in terms of auditing and testing had to be put in place. Often fraud is perpetrated in high value food commodities and those which come via complex supply chains. Probably herbs and spices fit these characteristics more than any other food ingredients and are thus highly vulnerable. Testing methods for the food industry must be easy to use, rapid and low costs. Our two tier system of testing provides not only a cost effective means of testing but one also that will survive rigours of a legal process. The survey data presented is disturbing in the level of adulteration found. It is clear that a serious level of fraud is being perpetrated and that bona fide businesses and consumers are being financially harmed. It is likely similar (if not worse) levels of fraud are occurring in many global regions. We believe the system we have developed and validated for oregano should be expanded to cover all herbs sold in the market. Only then will there be a sufficient deterrent in place to stop fraudulent activity in these widely consumed food ingredients.
